# Trends and correlates of driving under the influence of alcohol among different types of adult substance users in the United States: a national survey study

**DOI:** 10.1186/s12889-019-6889-8

**Published:** 2019-05-04

**Authors:** Ji-Yeun Park, Li-Tzy Wu

**Affiliations:** 1 Department of Psychiatry and Behavioral Sciences, BOX 3903, Duke University School of Medicine, Durham, NC USA; 2 Department of Medicine, Division of General Internal Medicine, Duke University School of Medicine, Durham, NC USA; 3 Duke Clinical Research Institute, Duke University School of Medicine, Durham, NC USA; 4 Center for Child and Family Policy, Sanford School of Public Policy, Duke University, Durham, NC USA

**Keywords:** Alcohol-impaired driving, Prevalence, Trends, Drug use type, Marijuana, Opioids, Polydrug use

## Abstract

**Background:**

Despite a decrease in driving under the influence of alcohol (DUIA) prevalence over the past decades, DUIA prevalence still remains high in the United States. To date, there is limited research examining whether different types of substance users have different trends in DUIA. This study sought to assess trends and variables associated with DUIA by substance use type.

**Methods:**

National Survey on Drug Use and Health (NSDUH) is a cross-sectional, nationally representative population-based survey. By using the NSDUH 2008–2014, we performed the Joinpoint analysis to identify time trends of DUIA in each group of substance users (aged ≥18 years). Logistic regression analysis was used to explore association between substance use type and DUIA and to identify variables associated with DUIA.

**Results:**

Adults who reported alcohol or drug use in the past year were classified into different groups based on past-year substance use status: alcohol use only (*n* = 141,521) and drug use regardless alcohol use. Drug users included prescription opioids only (*n* = 5337), marijuana only (*n* = 32,206), other single drug (*n* = 3789), prescription opioids-marijuana (*n* = 3921), multiple prescription drugs (*n* = 1267), and other multiple drugs (*n* = 18,432). The Joinpoint analysis showed that DUIA prevalence decreased significantly from 2008 to 2014 among alcohol only users (Average Annual Percent Change [AAPC] = − 2.8), prescription opioids only users (AAPC = -5.4), marijuana only users (AAPC = -5.0), prescription opioids-marijuana users (AAPC = -6.5), multiple prescription drug users (AAPC = -7.4), and other multiple drug users (AAPC = -3.2). Although the estimate was not statistically significant, other single drug users showed a decreasing trend (AAPC = -0.9). Substance use type was significantly associated with DUIA in the adjusted logistic regression. All drug use groups, relative to the alcohol only group, had elevated odds of DUIA, and the odds were especially elevated for the multiple drug use groups (prescription opioids-marijuana, adjusted odds ratio [AOR] = 2.71; multiple prescription drugs, AOR = 2.83; and other multiple drugs, AOR = 3.68). Additionally, younger age, male sex, being white, higher income, and alcohol abuse/dependence were positively associated with DUIA.

**Conclusions:**

DUIA prevalence decreased over time and the magnitude of this reduction differed by substance use type. DUIA interventions need to be tailored to substance use type and individual characteristics.

## Background

During the past few decades in the United States, the prevalence of driving under the influence of alcohol (DUIA) has decreased [1, 2]. Results from the National Survey on Drug Use and Health (NSDUH) showed that from 2005 to 2014, there was a 37% proportional decline in DUIA prevalence among US adults aged 18–24 years [3]. According to the 2013–2014 National Roadside Surveys of Alcohol and Drug Use by Drivers, the percentage of weekend nighttime drivers with breath alcohol concentration of .005–.049, .050–.079, and .08+ was 5.2, 1.6, and 1.5%, respectively; these percentages were remarkably lower as compared with 22.3, 6.1, and 7.5% in 1973 [4]. However, DUIA is still an important public health problem because prevalence rates remain high. According to results from the 2016 NSDUH, 8.2% of Americans aged 16 or older reported DUIA during the past year [5]. DUIA can pose a serious safety concern because alcohol is responsible for fatal crashes [6–8]. Alcohol-impaired driving crashes accounted for 28% of overall fatalities in the United States in 2016 [9]. Data from the US Department of Transportation Fatality Analysis Reporting System also showed that the prevalence of alcohol involvement in fatal crashes from 1999 to 2010 was at approximately 39% [10]. Economic costs associated with alcohol-involved crashes were estimated to be $52 billion [11].

Several studies examined the prevalence trends of DUIA in the United States by subgroup characteristics, such as race/ethnicity, sex, age, and drinking category [2, 12–15]; the prevalence of DUIA was higher among younger people, male, and people who reported binge drinking than their counterparts. However, population-based data on DUIA trends by substance use type are scarce. DUIA trends among marijuana users and prescription opioid misusers may be of particular interest because simultaneous or concurrent use of alcohol and these two drugs is commonly reported [16–19]. The use of both alcohol and marijuana was reported by 23% of US high school seniors who participated in the Monitoring the Future (MTF) study [20]. In another study using the MTF data, more than half of high school prescription opioid misusers reported use of prescription opioids and alcohol [21]. In the sample of motor vehicle crashes, marijuana was the most frequently detected non-alcohol substance [22]. The odds of fatal crashes increased significantly with combined use of alcohol and marijuana [23]. There is also evidence that fatal crashes among prescription opioid misusers is a growing concern. Between 1999 and 2000 and 2009–2010, the greatest increase in fatally injured drivers was associated with prescription narcotics; specifically, the prevalence of fatally injured drivers tested positive for hydrocodone/oxycodone increased over six times during the study period [24]. Marijuana and prescription opioids’ contribution to fatal crashes suggests a need for more research on DUIA trends by specific type of substance use.

Besides a single substance use, different types of multiple substance use also need to be taken into account when examining how DUIA trends differ by substance use type. The use of multiple substances was associated with greater probability of hazardous drinking that was found to be associated with increased risk of DUIA [25, 26]. A considerable body of evidence also suggests that multiple substance use was more strongly associated with fatal crashes than single substance use [27–29]. Marijuana and prescription opioids are the most commonly used non-alcohol substances in the United States [5]. Considering that marijuana use is associated with increased risk of prescription opioid misuse [30, 31], it is not surprising that a sizable proportion of prescription opioid misusers reported marijuana use [21, 32]. Despite the existing evidence that marijuana and prescription opioids are the most commonly detected non-alcohol substances in fatally injured drivers [33], DUIA trends among prescription opioids-marijuana users has been understudied. More attention also needs to be paid to the use of multiple prescription drugs as prescription drug misuse is widely considered a major public health issue. In a study of 3038 blood samples of suspected impaired drivers in Netherlands, 33% were positive for prescription drugs; of whom, 37% were multiple prescription drug users [34]. In addition, the use of multiple prescription drugs was found to be associated with increased crash risk [35].

Given the lack of research, more information on DUIA trends by single and multiple substance use would be useful in designing and implementing effective interventions to reduce DUIA. Accordingly, this study sought to examine trends and correlates of DUIA in the United States across different types of adult substance users.

## Methods

### Data

Data came from the 2008–2014 NSDUH public-use data files. The NSDUH is an annual cross-sectional survey supported by the Substance Abuse and Mental Health Services Administration. The NSDUH provides in-depth information on prevalence and patterns of substance use and substance use disorder in the civilian non-institutionalized population aged ≥12 years in the United States. The target population of NSDUH includes residents of households, non-institutional group quarters (e.g., shelters, rooming houses, dormitories), and civilian living on a military base. Individuals living in institutional group quarters (e.g., jails and hospitals), active duty military personnel, and individuals with no fixed address (e.g., homeless) were not included in the NSDUH. In the NSDUH, data were collected using computer-assisted interviewing methods. To ensure accuracy, data regarding private and confidential questions (e.g., illicit drug use, mental health, and other sensitive behaviors) were collected using audio computer-assisted self-interviewing [36]. For less sensitive demographic items, computer-assisted personal interviewing was used. The NSDUH employed a multistage area probability sample in each of the 50 states and the District of Columbia. Weighted screening and response rates for the 2008–2014 NSDUH were 81.9–88.8% and 71.2–74.7%, respectively.

In this study, we focused on adults (aged ≥18 years) who reported alcohol or drug use in the past year. Adult substance users include “alcohol only users (non-drug use)” and “one or more drug users regardless of alcohol use status.” The latter group was further classified into six groups: prescription opioids only; marijuana only; other single drug; prescription opioids-marijuana; multiple prescription drugs; and other multiple drugs. A total of 206,473 adult substance users (aged ≥18 years) from the 2008–2014 NSDUH were analyzed.

### Study variables

#### Drug use in the past year

NSDUH included information on alcohol and drug use from nine drug categories: marijuana, cocaine, heroin, hallucinogens, inhalants, as well as misuse of prescription pain relievers, tranquilizers, stimulants, and sedatives. In the NSDUH, misuse of prescription drugs was defined as use of medication without a prescription or use only for the experience or feeling caused by medication. Respondents who reported lifetime use of each drug (marijuana, cocaine, heroin, hallucinogens, inhalants, prescription pain relievers, tranquilizers, stimulants, and sedatives) were asked about recency of use. Individuals were considered as past-year substance user if they used alcohol or drug within the past 12 months.

#### Driving under the influence of alcohol in the past year

The NSDUH asked respondents, (a) “During the past 12 months, have you driven a vehicle while you were under the influence of a combination of alcohol and illegal drugs used together?” and (b) “During the past 12 months, have you driven a vehicle while you were under the influence of alcohol only?” In the NSUDH, DUIA was defined as positive response to either of the questions (a) or (b).

#### Alcohol abuse and dependence in the past year

Alcohol abuse and alcohol dependence were included in the analysis as covariates given a possible association with DUIA [37, 38]. The NSDUH used DSM-IV criteria for substance abuse and dependence [39]. Based on the DSM-IV criteria, those who reported at least one of four alcohol abuse symptoms but did not meet the criteria for alcohol dependence during the past year were considered as having alcohol abuse. Those who reported at least three out of seven alcohol dependence symptoms during the past year were considered as having alcohol dependence.

#### Sociodemographic characteristics

Sociodemographic characteristics included age (18–25, 26–34, 35–49, 50+), sex (male and female), race/ethnicity (non-Hispanic White, non-Hispanic Black, Hispanic, Asian/Native Hawaiian/Other Pacific Islander, and other), annual household income (≤$49,999, $50,000–$74,999, and ≥ $75,000), and population density (large metro, small metro, and non-metro). These sociodemographic characteristics were included as covariates in the analysis based on prior findings that indicated a possible association with DUIA [40–43]. Survey year was included as a categorical variable in the descriptive statistics to produce the prevalence of DUIA by year. In the adjusted analysis, survey year was considered as a continuous variable to explore time trends of DUIA.

### Statistical analysis

First, we conducted a descriptive analysis to examine distributions of sociodemographic characteristics across seven different types of adult substance users. We then examined past-year prevalence of DUIA by substance use type. To identify statistically significant changes in DUIA trend over time in each group, the Joinpoint analysis was performed [44]. The Joinpoint analysis was used to identify time points where a statistically significant change in linear slope of the trend occurred. The analysis started with zero joinpoints (a straight line) and assessed model fit by adding up to a maximum number of joinpoints. In the final model, the best-fitting joinpoints were selected [45]. Joinpoint analysis also estimated annual percentage change (APC) and average annual percent change (AAPC) [45]. Finally, logistic regression analysis was performed to explore association between DUIA and substance use type (reference group = alcohol only), adjusting for age, race/ethnicity, sex, annual household income, population density, past-year alcohol abuse/dependence, and survey year. Variables associated with DUIA were also examined using adjusted logistic regression. We reported 95% confidence intervals (CIs) and adjusted odds ratios (ORs) to help interpretation. All results, except for sample size, were weighted to account for the NSDUH’s complex survey designs, such as weighting and clustering effects. All statistical analyses, except the Joinpoint analysis, were performed using SAS 9.4 (Cary, NC). The Joinpoint analysis was performed using the Joinpoint software version 4.7.0.0 (National Cancer Institute, 2019). This study was exempted from the Duke University Health System Institutional Review Board due to use of publicly available, de-identification datasets.

## Results

### Sociodemographic (Table 1)

Among all adult substance users (*N* = 206,473), 78.80% (*n* = 141,521) were alcohol only users (non-drug use) and the remaining were drug users regardless of alcohol use status. The proportions of drug use groups were as follows: prescription opioids only (2.31%, *n* = 5337), marijuana only (10.47%, *n* = 32,206), other single drug (1.65%, *n* = 3789), prescription opioids-marijuana (1.08%, *n* = 3921), multiple prescription drugs (0.55%, *n* = 1267), and other multiple drugs (5.14%, *n* = 18,432). Past-year alcohol abuse and dependence were much prevalent among multiple drug users (alcohol abuse; 17.36% and alcohol dependence; 19.71%) than among alcohol only users (alcohol abuse; 3.57% and alcohol dependence; 2.93%).

**Table 1:** Sociodemographic characteristics of adults who reported alcohol or drug use in the past year, NSDUH 2008–2014

		Adult substance users (N = 206,473)						
	Total	Alcohol only	Prescription opioids only^a^	Marijuana only^a^	Other single drug^a^	Prescription opioids-Marijuana^a^	Multiple prescription drugs^a^	Other multiple drugs^a^
Sample size	N = 206,473	n = 141,521 (78.80%)	n = 5337 (2.31%)	n = 32,206 (10.47%)	n = 3789 (1.65%)	n = 3921 (1.08%)	n = 1267 (0.55%)	n = 18,432 (5.14%)
	N (%)	N (%)	N (%)	N (%)	N (%)	N (%)	N (%)	N (%)
Age in years								
18–25	100,189 (16.28)	56,075 (11.40)	2829 (19.19)	21,723 (34.22)	1999 (20.11)	2878 (42.35)	596 (18.18)	14,089 (46.38)
26–34	33,909 (17.80)	24,313 (16.16)	965 (22.63)	4651 (22.87)	669 (21.95)	583 (26.74)	268 (26.51)	2460 (26.29)
35–49	44,233 (28.38)	36,100 (29.67)	1099 (32.73)	4118 (23.19)	774 (30.89)	361 (19.82)	293 (32.03)	1488 (17.89)
50 +	28,142 (37.54)	25,033 (42.77)	444 (25.44)	1714 (19.73)	347 (27.05)	99 (11.09)	110 (23.28)	395 (9.43)
Sex								
Male	100,107 (51.03)	64,513 (49.08)	2387 (49.37)	17,803 (59.83)	1721 (47.54)	2328 (63.00)	442 (40.00)	10,913 (63.43)
Female	106,366 (48.97)	77,008 (50.92)	2950 (50.63)	14,403 (40.17)	2068 (52.46)	1593 (37.00)	825 (60.00)	7519 (36.57)
Race/ethnicity								
Non-Hispanic White	136,140 (70.65)	94,604 (71.33)	3148 (61.68)	19,281 (65.83)	2449 (70.42)	2566 (71.40)	923 (75.65)	13,169 (73.39)
Non-Hispanic Black	23,657 (10.47)	14,991 (9.80)	695 (12.08)	5671 (16.37)	322 (7.79)	541 (13.23)	89 (7.05)	1348 (8.61)
Hispanic	29,583 (13.05)	20,622 (12.96)	993 (18.83)	4279 (12.24)	667 (16.51)	445 (10.14)	171 (14.15)	2406 (12.89)
Asian/Native Hawaiian/Pacific Islander	7882 (4.05)	6113 (4.41)	235 (5.09)	873 (2.57)	149 (2.71)	82 (1.91)	28 (2.00)	402 (2.22)
Other	9211 (1.78)	5191 (1.50)	266 (2.33)	2102 (2.99)	202 (2.57)	287 (3.32)	56 (1.15)	1107 (2.89)
Annual household income								
≤$49,999	116,601 (45.48)	73,790 (42.03)	3393 (53.87)	21,299 (57.51)	2268 (53.68)	2673 (62.81)	773 (55.02)	12,405 (62.90)
$50,000–$74,999	33,718 (17.82)	24,874 (18.54)	816 (18.00)	4339 (15.07)	573 (16.41)	574 (15.14)	192 (15.00)	2350 (13.52)
≥$75,000	56,154 (36.70)	42,857 (39.43)	1128 (28.13)	6568 (27.42)	948 (29.91)	674 (22.05)	302 (29.98)	3677 (23.58)
Population density								
Large metro	92,298 (55.33)	62,387 (55.00)	2319 (54.74)	15,154 (57.50)	1793 (56.49)	1680 (52.82)	529 (49.72)	8436 (57.06)
Small metro	72,108 (30.00)	49,114 (29.94)	1950 (30.38)	11,106 (29.60)	1340 (30.62)	1412 (31.10)	476 (34.33)	6710 (30.69)
Non-metro	42,067 (14.67)	30,020 (15.06)	1068 (14.88)	5946 (12.90)	656 (12.89)	829 (16.08)	262 (15.95)	3286 (12.25)
Past-year alcohol abuse/dependence								
None	177,574 (89.95)	129,606 (93.50)	4449 (85.29)	25,794 (81.51)	3000 (82.04)	2672 (69.37)	955 (77.32)	11,098 (62.93)
Abuse	15,738 (5.29)	6889 (3.57)	460 (7.40)	3606 (10.05)	380 (7.93)	669 (16.35)	134 (9.11)	3600 (17.36)
Dependence	13,161 (4.76)	5026 (2.93)	428 (7.31)	2806 (8.44)	409 (10.03)	580 (14.28)	178 (13.57)	3734 (19.71)
Survey Year								
2008	28,441 (13.66)	19,715 (13.95)	900 (15.95)	3797 (11.10)	582 (14.72)	567 (12.35)	199 (12.04)	2681 (13.44)
2009	28,952 (14.03)	19,695 (14.16)	826 (13.92)	4206 (12.39)	532 (13.84)	633 (15.81)	195 (15.79)	2865 (14.87)
2010	29,978 (14.07)	20,474 (14.11)	794 (14.79)	4539 (13.40)	542 (13.15)	589 (13.99)	222 (17.19)	2818 (14.45)
2011	29,743 (14.16)	20,284 (14.34)	767 (12.89)	4728 (14.05)	548 (13.28)	633 (14.66)	148 (12.24)	2635 (12.65)
2012	28,963 (14.54)	19,461 (14.39)	752 (15.81)	4841 (14.93)	527 (14.51)	566 (13.78)	187 (17.70)	2629 (15.31)
2013	28,667 (14.62)	19,378 (14.47)	662 (13.66)	4961 (15.92)	503 (15.08)	493 (15.70)	150 (11.65)	2520 (14.74)
2014	31,729 (14.93)	22,514 (14.59)	636 (12.97)	5134 (18.22)	555 (15.42)	440 (13.72)	166 (13.39)	2284 (14.54)

Sample size is unweight, and results are weighted estimates

*CI* Confidence Interval

^a^ Past-year drug use, regardless of pas-year alcohol use status

### Prevalence trends of past-year DUIA in the United States, 2008–2014 (Table 2)

Overall, other multiple drug users had the highest prevalence of past-year DUIA (48.30, 95% CI: 47.01–49.60), followed by prescription opioids-marijuana users (39.37, 95% CI: 37.12–41.63), multiple prescription drug users (34.70, 95% CI: 30.79–38.61), marijuana only users (29.10, 95% CI: 28.13–30.07), other single drug users (27.50, 95% CI: 25.43–29.57), and prescription opioids only users (22.16, 95% CI: 20.20–24.13). Alcohol only users had the lowest prevalence of DUIA (12.93, 95% CI; 12.65–13.21). Table 2 also displays the results of the joinpoint regression analysis. The final model selected zero joinpoints for all substance use groups, except other single drug users (one joinpoint in 2012) from 2008 to 2014. During the study period, there was significant reduction in DUIA prevalence among alcohol only users (AAPC = − 2.8, 95% CI: -4.7, − 0.9), prescription opioids only users (AAPC = − 5.4, 95% CI: -9.5, − 1.1), marijuana only users (AAPC = − 5.0, 95% CI: -5.8, − 4.1), prescription opioids-marijuana users (AAPC = − 6.5, 95% CI: -8.8, − 4.0), multiple prescription drug users (AAPC = -7.4, 95% CI: -13.6, − 0.8), and other multiple drug users (AAPC = − 3.2, 95% CI: -5.0, − 1.3). The final model selected for DUIA among other single drug users detected one joinpoint in 2012. Although the estimates were not statistically significant, DUIA prevalence among other single drug users increased by 8.7% per year (APC = 8.7, 95% CI: -13.2, 36.2) from 2008 to 2012 and then decreased by 17.6% per year (APC = -17.6, 95% CI: -57.2, 58.8) from 2012 to 2014. The AAPC was − 0.9 (95% CI: -12.2, 11.9) for other single drug users, indicating that from 2008 to 2014, on average, DUIA decreased by 0.9% per year.

**Table 2:** Trends of driving under the influence of alcohol: by substance use type, United States, NSDUH 2008–2014

	Past-year alcohol use	Past-year drug use, regardless of pas-year alcohol use status					
	Alcohol only	Prescription opioids only	Marijuana only	Other single drug	Prescription opioids-Marijuana	Multiple prescription drugs	Other multiple drugs
Sample size	*n* = 139,835	*n* = 5224	*n* = 31,982	*n* = 3710	*n* = 3903	*n* = 1262	n = 18,367
	% (95% CI)	% (95% CI)	% (95% CI)	% (95% CI)	% (95% CI)	% (95% CI)	% (95% CI)
Overall prevalence	12.93 (12.65–13.21)	22.16 (20.20–24.13)	29.10 (28.13–30.07)	27.50 (25.43–29.57)	39.37 (37.12–41.63)	34.70 (30.79–38.61)	48.30 (47.01–49.60)
Year 2008	14.63 (13.74–15.52)	28.69 (23.29–34.10)	34.57 (30.86–38.29)	24.27 (18.34–30.21)	46.20 (40.82–51.58)	45.24 (35.93–54.55)	53.60 (50.77–56.44)
Year 2009	13.28 (12.52–14.04)	22.58 (18.14–27.02)	32.89 (30.09–35.69)	24.25 (18.14–30.37)	46.81 (40.36–53.27)	37.38 (24.89–49.87)	52.42 (48.97–55.87)
Year 2010	12.65 (11.90–13.41)	21.73 (16.89–26.57)	30.62 (27.80–33.44)	32.19 (25.20–39.18)	42.89 (35.98–49.80)	38.91 (29.09–48.73)	49.18 (46.01–52.35)
Year 2011	13.39 (12.53–14.24)	23.63 (17.35–29.91)	28.54 (26.51–30.57)	29.04 (22.38–35.69)	39.28 (33.78–44.78)	25.45 (14.50–36.41)	46.63 (43.73–49.54)
Year 2012	12.22 (11.50–12.94)	18.30 (13.90–22.69)	28.30 (25.81–30.80)	34.77 (27.67–41.87)	35.58 (30.05–41.12)	34.32 (24.28–44.37)	44.72 (41.11–48.33)
Year 2013	12.70 (11.93–13.47)	19.90 (14.32–25.49)	26.85 (24.64–29.06)	25.56 (18.75–32.36)	31.30 (25.35–37.24)	36.94 (25.45–48.42)	48.62 (44.97–52.26)
Year 2014	11.71 (11.07–12.35)	19.72 (15.91–23.52)	25.14 (23.42–26.86)	23.29 (19.68–26.91)	34.21 (27.44–40.97)	23.67 (15.75–31.58)	43.21 (39.64–46.79)
Joinpoints^a^							
	Zero joinpoints	Zero joinpoints	Zero joinpoints	One joinpoint (in 2012)	Zero joinpoints	Zero joinpoints	Zero joinpoints
Annual percent change (APC)^b^							
Before joinpoint	−2.8* (−4.7, −0.9)	−5.4* (−9.5, −1.1)	−5.0* (−5.8, −4.1)	8.7 (−13.2, 36.2)	−6.5* (−8.8, − 4.0)	−7.4* (− 13.6, − 0.8)	−3.2* (− 5.0, − 1.3)
After joinpoint	−2.8* (− 4.7, − 0.9)	− 5.4* (− 9.5, − 1.1)	−5.0* (− 5.8, − 4.1)	−17.6 (− 57.2, 58,8)	−6.5* (− 8.8,-4.0)	− 7.4* (− 13.6, − 0.8)	−3.2* (− 5.0, − 1.3)
Average annual percent change (AAPC)^c^							
2008–2014	− 2.8* (− 4.7, − 0.9)	−5.4* (− 9.5, − 1.1)	−5.0* (− 5.8, − 4.1)	−0.9 (− 12.2, 11.9)	− 6.5* (− 8.8,-4.0)	−7.4* (− 13.6, − 0.8)	−3.2* (− 5.0, − 1.3)

*CI* Confidence Interval

* Statistically significantly different from zero (*p* < .05)

^a^ Joinpoints were identified with joinpoint regression. The final model selected by joinpoint regression was used

^b^ Annual percent change was estimated by joinpoint regression. APC was the same before and after joinpoint when zero joinpoints were detected

^c^ APC and AAPC were the same when zero joinpoints were detected

Note. Sample size varied due to responses with unknown values

### Adjusted logistic regression model predicting variables associated with DUIA (Table 3)

Table 3 displays the results of the adjusted logistic regression analysis. The odds of DUIA varied by substance use type. Compared with alcohol only users, all other substance use groups had increased odds of DUIA. Prescription opioids only users were 1.65 times (95% CI: 1.47–1.86) more likely than alcohol only users to report DUIA. Marijuana only (95% CI: 2.04–2.32) and other single drug users (95% CI: 1.88–2.39) had nearly 2 times higher odds of DUIA than alcohol only users, respectively. Using two or more drugs was strongly associated with DUIA. The ORs showed a dose related pattern with multiple drug use groups having greater odds of DUIA than the group either using prescription opioids only or using marijuana only. Compared with alcohol only users, prescription opioids-marijuana and multiple prescription drug users had 2.71 times (95% CI: 2.43–3.02) and 2.83 times (95% CI: 2.35–3.41) higher odds of DUIA, respectively. Other multiple drug users had the highest odds of DUIA (adjusted OR = 3.68, 95% CI: 3.40–3.97). Younger age, male sex, being white, higher income, and alcohol abuse/dependence were significant variables associated with DUIA. Survey year was found to be a significant predictor in the adjusted analysis, indicating a decreasing trend of DUIA from 2008 to 2014.

**Table 3:** _Adjusted logistic regression model predicting variables associated with driving under the influence of alcohol_

	Adjusted OR (95% CI) (*N* = 204,283)
Age	
18–25 (ref.)	1.00
26–34	**1.22 (1.17–1.28)**
35–49	0.95 (0.90–1.00)
50+	**0.65 (0.62–0.69)**
Sex	
Male	**1.60 (1.53–1.66)**
Female (ref.)	1.00
Race/ethnicity	
Non-Hispanic White	**1.46 (1.35–1.58)**
Non-Hispanic Black (ref.)	1.00
Hispanic	0.94 (0.86–1.03)
Asian/Native Hawaiian/Pacific Islander	**0.72 (0.62–0.83)**
Other	1.04 (0.90–1.21)
Annual household income	
≤$49,999 (ref.)	1.00
$50,000–$74,999	**1.55 (1.46–1.64)**
≥$75,000	**1.80 (1.72–1.88)**
Population density	
Large metro	1.06 (1.00–1.12)
Small metro	**1.10 (1.04–1.17)**
Non metro (ref.)	1.00
Alcohol abuse/dependence (past-year)	
None (ref.)	1.00
Abuse	**9.44 (8.79–10.14)**
Dependence	**5.99 (5.54–6.47)**
Year	**0.97 (0.96–0.98)**
Substance use type	
Alcohol only (ref.)	1.00
Prescription opioids only ^a^	**1.65 (1.47–1.86)**
Marijuana only ^a^	**2.18 (2.04–2.32)**
Other single drug ^a^	**2.12 (1.88–2.39)**
Prescription opioids-marijuana ^a^	**2.71 (2.43–3.02)**
Multiple prescription drugs ^a^	**2.83 (2.35–3.41)**
Other multiple drugs ^a^	**3.68 (3.40–3.97)**

*OR* Odds Ratio, *CI* Confidence Interval, *Ref*. reference group

^a^ Past-year drug use, regardless of pas-year alcohol use status

Boldface: *p* < .01

## Discussion

This study examined changes in DUIA trend by substance use type and identified variables associated with DUIA. By using the joinpoint analysis, we estimated the APC and AAPC in each group of substance users over the study period (2008–2014). The largest decline was observed among multiple prescription drug users with the AAPC of − 7.4%. The AAPC was − 6.5% among prescription opioids-marijuana users, − 5.4% among prescription opioids only users, − 5.0% among marijuana only users, − 3.2% among other multiple drug users, and − 2.8% among alcohol only users. Single drug users showed the smallest decline (AAPC = − 0.9%) from 2008 to 2014, which was not statistically significant. DUIA prevalence among other single drug users increased from 2008 to 2012 (APC = 8.7%) and then decreased from 2012 to 2012 (APC = − 17.6%). Such decreasing trends of DUIA across all substance use groups, regardless of statistical significance, suggest that national efforts may have reduced DUIA to some extent. However, the estimated prevalence of DUIA among adult substance users still remains considerably high (ranging from 12.93 to 48.30%, overall) and the magnitude of decline in DUIA prevalence differed by substance use type. Clearly, there is a continuing need for prevention efforts to reduce DUIA among all substance use groups at the national level.

In recent years, there has been a significant change in laws surrounding prescription opioids and marijuana, such as prescription drug monitoring program (PDMP) and marijuana legalization [46, 47]. Thus, it is necessary to further assess the impacts of such laws on DUIA. PDMP is a state-level database that tracks controlled substance prescriptions. Studies have reported some positive effects of PDMP on reducing prescription drug misuse [48–50]. Considering the high probability of use of both alcohol and prescription drugs [16, 17, 21, 51], it would be important to assess long-term impacts of PDMP on the DUIA occurrence as well as prescription drug misuse. State marijuana legalization laws also have changed over time in the United States. Available data have suggested that marijuana legalization appeared to contribute to the reduction in alcohol consumption [52, 53]. However, current evidence is insufficient to determine how marijuana legalization could influence alcohol impaired driving [54]. Given the lack of evidence, additional research is needed to understand the mechanism underlying the effects of policy intervention, such as PDMP and marijuana legalization, on DUIA among subtypes of prescription opioids and marijuana users.

This study also found that the strength of associations with DUIA varied by substance use type. Prescription opioids only users had 1.65 times higher odds of DUIA than alcohol only users. This may be due to a higher prevalence of alcohol abuse/dependence among prescription opioids only users than alcohol only users in this sample, and alcohol abuse/dependence was positively associated with DUIA in the adjusted analysis. A previous study also found that prescription opioid misuse was more prevalent among people with alcohol use disorder than people without alcohol use disorder [55]. Marijuana and other single drug users also had increased odds of DUIA than alcohol only users. Using two or more drugs was strongly associated with DUIA, which is consistent with previous research examining association of being arrest for driving under the influence [56]. Of different types of multiple drug use, the highest adjusted OR was associated with other multiple drug use (adjusted OR = 3.68), followed by multiple prescription drugs (adjusted OR = 2.83) and prescription opioids-marijuana (adjusted OR = 2.71). When designing and implementing DUIA prevention programs, it is important to identify high-risk groups of DUIA, such as multiple drugs users with alcohol use disorder, so that they can receive intensive interventions. Several studies have documented the effectiveness of a social norm strategy in reducing impaired driving [57, 58]. Based on our study findings, social norms interventions should be implemented by addressing substance use pattern in order to improve their effectiveness. For example, multiple drug users could be exposed to the social norms interventions more frequently (e.g., increasing duration or number of sessions) than alcohol only or single drug users.

This study also revealed a number of variables associated with DUIA. Younger age, male sex, being White, higher income, and alcohol abuse/dependence were significant predictors of DUIA. Interestingly, higher-income people were more likely to report DUIA than lower-income people. A previous study found that higher-income people tended to have higher alcohol consumption [59], which may explain the positive association between higher-income and DUIA. In light of these findings, consistent monitoring and education programs should be provided, particularly for young white males. Alcohol abuse/dependence were the most powerful predictors of DUIA. Alcohol abuse/dependence were also more prevalent among drug use groups than alcohol use only group in this study sample, which might attributable to the elevated odds of DUIA among all other substance use groups, compared with alcohol only use group. Such findings underscore the importance of DUIA prevention programs targeting alcohol and other substance users, especially those with alcohol use disorder. As part of DUIA preventive efforts, screening and treatment for alcohol and drug use disorders should be implemented in clinical settings [60, 61]. Alcohol consumption monitoring also appeared to be effective in preventing impaired driving [62]. Consistent monitoring of alcohol consumption and drug use, particularly for people with a substance use disorder, would play a vital role in preventing DUIA.

This study has some limitations. First, this study could not establish temporal or causal relationship due to the nature of the cross-sectional data. Future longitudinal studies are needed to explore the extent to which different types of substance use lead to DUIA. In addition, the prevalence of DUIA was estimated based on self-reports, which might lead to biased prevalence estimations. It is possible that respondents did not report correctly due to social stigma associated with DUIA. Additional research investigating the prevalence of DUIA from oral fluid and/or blood samples may provide a more accurate estimate. Lastly, this study focused on self-reported use of one or more drugs in the past year. The precise timing of use could not be determined from the data source. Previous studies showed that simultaneous use of multiple drugs was more strongly associated with alcohol-related problems as well as unsafe driving than concurrent use of multiple drugs [16, 63]. Thus, examining differences in DUIA risk between simultaneous and concurrent users of multiple drugs would be of interest for further research.

## Conclusions

This study demonstrated different time trends of DUIA by substance use type. From 2008 to 2014, DUIA prevalence decreased significantly in all substance use groups, except for other single drug users, with the largest reduction noted among multiple prescription drug users. Although the estimate was not statistically significant, single drug users also showed a decreasing trend of DUIA. Our results lend support in implementing DUIA prevention interventions that should be tailored to substance use type to more effectively reduce DUIA. Furthermore, the strength of associations with DUIA varied by substance use type. In particular, using two or more drugs yielded the highest odds of DUIA, indicating a need for more intensive DUIA intervention programs targeting multiple drug users. Younger people, male, white (vs. black), those with higher income (vs. those with household incomes of less than $49,999), and people with alcohol use disorder were also more likely to report DUIA. Our findings highlight the importance of enhanced DUIA interventions by adding individual sessions that focus on high-risk subgroups [64], such as multiple drug users and individuals with the above characteristics. Additionally, policy makers should take into account the variations in the likelihood of DUIA by substance use type when designing and implementing strategies for reducing DUIA. Further research is warranted to clarify whether simultaneous use of multiple drugs leads to higher risk of DUIA than concurrent use of multiple drugs.

## Abbreviations

AAPC: Average annual percent change
APC: Annual percent change
CI: Confidence interval
DUIA: Driving under the influence of alcohol
MTF: Monitoring the Future
NSDUH: National Survey on Drug Use and Health
OR: Odds ratio
PDMP: Prescription drug monitoring program
